# Supplementary material for: Incorporating the speciation process into species delimitation
Source: PLoS Comput Biol. 2021 May 13;17(5):e1008924. doi: 10.1371/journal.pcbi.1008924 (PMC8118268; doi:10.1371/journal.pcbi.1008924)
Supplement: S1 Text — (PDF) [file pcbi.1008924.s001.pdf]

Description of the DELINEATE algorithm

Let N represent the number of tips in the tree. DELINEATE calculates the probability of a particular partition via traversing the a tree from tip to root. The calculations can be described as filling in a pair of three-dimensional look-up tables of probabilities, d and a, which are indexed by (1) a node index, (2) a partition index vector, and (3) an open subset index.

At the end of the algorithm $d[i][\mathbf{j}][k]$ would store the probability of the species partition $\mathbf{j}$ evolving on the subtree rooted at node i with the node i conspecific with subset k in the partition. The look-up table a is indexed similarly, but it holds the probabilities for the root-most portion of the lineage that ends in node i.

The domain of the node index (denoted i unless otherwise stated) is $[1, 2, \ldots 2N-1]$ where indices $[1, 2, \ldots N]$ are the indices for the N tips of the tree, and the indices $[N+1, N+2, \ldots 2N-1]$ uniquely identify the internal nodes of the tree, with the largest value corresponding to the root.

The domain of the partition index (denoted $\mathbf{j}$ unless otherwise stated) is the set of all N-dimensional vectors where the elements have values in the range $[0, 1, 2, \ldots N]$. The value of the i-th element of this vector holds index of the subset node i is a member of, where 0 is used to indicate that node i is not in the subtree that is covered by this paritition. The ordering of the indices for the subsets is arbitrary, but for convenience, we will assume that if the partition has x non-empty subsets, then the indices for them will be $[1, 2, \ldots x]$.

The subset index (denoted k unless otherwise stated) indicates which (if any) subset of the partition is conspecific with the part of the lineage for node i which is the first dimension in the look-up table. Here $k = 0$ indicates that the specified portion of the lineage is not conspecific with any subset of the tips descended from the node.

In practice, one can use a data structure that can contain missing entries rather than filling in the entire look-up table. We can rely on being able to efficiently iterate over the non-missing elements of the look-up table, and treat other elements as holding probabilities of 0.0 Let $\mathcal{V}(i)$ denote the set of all partition indices that have non-zero values for node i. In other words, $\mathcal{V}(i)$ is the set of values for $\mathbf{j}$ for which $d[i][\mathbf{j}][k] > 0$ for some k.

The calculations proceed in a traversal (such as a postorder traversal) in which descendant nodes are visited before their ancestors. At each step i is the index of the current node in the traversal.

Tip initialization of elements of d

If node i is a leaf:

$$d[i][\mathbf{j}][k] = \begin{cases} 1 & \text{if } k = 1 \text{ and } j_i = 1 \text{ and } j_m = 0 \ \forall m \neq i \\ 0 & \text{otherwise} \end{cases} \tag{1}$$

This initialization simply states that leaf i is conspecific with itself and is not the ancestor of any other node. In practice, one can simply fill in the one element that has a probability of 1 and leave the other portions of $d[i]$ empty.

Calculation of elements of a for tips or internal nodes

The only evolutionary event that can occur along a branch is a speciation completion event. This only effects scenarios in which the descendant node was conspecific with a nonempty set of tips.

As noted in the text, if τ_i is the duration of the branch leading to node i and σ is the speciation completion rate then:

$$c_i = 1 - e^{-\sigma \tau_i} \tag{2}$$

is the probability of at least one speciation completion event occurring on the branch leading to i. To fill in elements of the root-ward look-up table for lineage i we visit every non-empty partition index $\mathbf{j} \in \mathcal{V}(i)$ and for each valid $k > 0$ we set:

$$a[i][\mathbf{j}][k] = (1 - c)d[i][\mathbf{j}][k] \tag{3}$$

indicating that the probability of the oldest part of the lineage i being conspecific with some tips depends on no speciations completion event having occured. To fill in the values associated with the root-most part lineage i not being conspecific with any tip, we use:

$$a[i][\mathbf{j}][0] = d[i][\mathbf{j}][0] + c \sum_{m=1}^{k} d[i][\mathbf{j}][k] \tag{4}$$

to reflect that the possibilities that the tip-most portion of linage i was not conspecific with any tip or at least one speciation completion event occurred along branch i.

Internal node calculations for d

If node i is an internal node, the values for $d[i][][]$ are constructed from its daughter nodes; let l_i and r_i be the indices for the left and right daughter nodes of node i. The model does not allow for any speciation completion at lineage splitting events. Thus, any partition index $\mathbf{j}$ and subset index $k > 0$ with a probability greater than 0 stored in $a[l_i][\mathbf{j}][k]$ or $a[r_i][\mathbf{j}][k]$ corresponds to partitions that will contribute a non-zero probability in $d[i][\mathbf{x}][k]$ for some partition index $\mathbf{x}$.

Note that in the partition indexing notation we are using, indices corresponding to leaves that descend from a node will have values greater than 0 in the partition indexing vector, and all leaves not descended from the node will have 0 elements in the vector. Since the descendants of internal node i are the union of the descendants of nodes l_i and r_i. Thus the partition index vectors holding non-zero probabilities for node i will be a distinct set of indices from those of the daughter nodes.

If we consider all combinations of partitions indices $\mathbf{s} \in \mathcal{V}(l_i)$ and $\mathbf{r} \in \mathcal{V}(r_i)$, we must consider how the partitions merge into a partition of leaves in the ancestor.

Case in which the internal node is conspecific with some descendants of each daughter node

Consider a combination for which $a[l_i][\mathbf{s}][k_l] > 0$ and $a[r_i][\mathbf{r}][k_r] > 0$ and for which $k_l > 0$ and $k_r > 0$. When filling in the ancestral value, we have to create a partition index which indicates that subset k_l of $\mathbf{s}$ is conspecific with k_r of r, and both are conspecific with the internal node i. Multiple possible conventions could be used. Let $\max_{\mathbf{j}}$ denote the largest element in partition index vector $\mathbf{j}$. Without loss of generality we could define the parental partition index, $\mathbf{x}$ to have the following values for its N elements:

$$x_m = \begin{cases} 0 & \text{if } s_m = 0 \text{ and } r_m = 0 \\ s_m & \text{if } s_m > 0 \\ \max_{\mathbf{s}} + r_m & \text{if } 0 < r_m < k_r \\ k_l & \text{if } r_m = k_r \\ \max_{\mathbf{s}} + r_m - 1 & \text{if } r_m > k_r \end{cases} \tag{5}$$

which guarantees that the subset indexing scheme for the left child is used for the first $\max_{\mathbf{s}}$ indices, but the subset indices from the right child are increased to avoid collision of subset indices with the exception that subset k_r must be conspecific to the subset k_l from the left child. The "minus 1" in the final case, merely keeps the maximum number in the parental index as small as possible by accounting for the merged subsets of taxa that are conspecific to the ancestor.

Given this definition of $\mathbf{x}$, we can fill in this element of the internal node look-up table as the product:

$$d[i][\mathbf{x}][k_l] = (a[l_i][\mathbf{s}][k_l])\,(a[r_i][\mathbf{r}][k_r]) \tag{6}$$

Case in which the internal node is not conspecific descendants of both daughter nodes

It is simpler to generate the internal node's partition index, $\mathbf{x}$, in the case of combination in which at least one of k_r and k_l are non-zero. If $k_r = 0$, we define $\mathbf{x}$ as:

$$x_m = \begin{cases} s_m & \text{if } r_m = 0 \\ \max_{\mathbf{s}} + r_m & \text{otherwise} \end{cases} \tag{7}$$

and fill in the internal node look-up table using:

$$d[i][\mathbf{x}][k_l] = (a[l_i][\mathbf{s}][k_l])\,(a[r_i][\mathbf{r}][0])\,. \tag{8}$$

Note that this includes cases in which $k_l = 0$.

Similarly, if $k_l = 0$, but $k_r > 0$, we define $\mathbf{x}$ as:

$$x_m = \begin{cases} r_m & \text{if } s_m = 0 \\ \max_{\mathbf{r}} + s_m & \text{otherwise} \end{cases} \tag{9}$$

and fill in the internal node look-up table using:

$$d[i][\mathbf{x}][k_r] = (a[l_i][\mathbf{s}][0])\,(a[r_i][\mathbf{r}][k_r])\,. \tag{10}$$

Note that this case excludes $k_r = 0$, because must avoid double-counting by assigning cases in which $k_l = k_r = 0$ to two distinct partitions.

Finalization

At the end of the traversal the probabilities for all of the partitions are stored in $d[2N-1][\mathbf{j}][k]$. One may skip the calculation of $a[2N-1][\mathbf{j}][k]$, as that portion of the look-up table is not used.

We are interested in the partition of leaves into species, regardless of whether any species is conspecific with the root node. Thus we can store the final probabilities for each partition in an array, f, indexed by partition index vectors, $\mathbf{j}$. For every $\mathbf{j} \in \mathcal{V}(2N-1)$:

$$f[\mathbf{j}] = \sum_{k=0}^{\max \mathbf{j}} d[2N-1][\mathbf{j}][k]. \tag{11}$$
