# Supplementary figures and images for: Incorporating the speciation process into species delimitation
Source: PLoS Comput Biol. 2021 May 13;17(5):e1008924. doi: 10.1371/journal.pcbi.1008924 (PMC8118268; doi:10.1371/journal.pcbi.1008924)

Estimated Speciation Completion Rate

0.175  
0.150  
0.125  
0.100  
0.075  
0.050  
0.025  
0.000

0.001  
0.004  
0.01

0.04

0.1

True Speciation Completion Rate

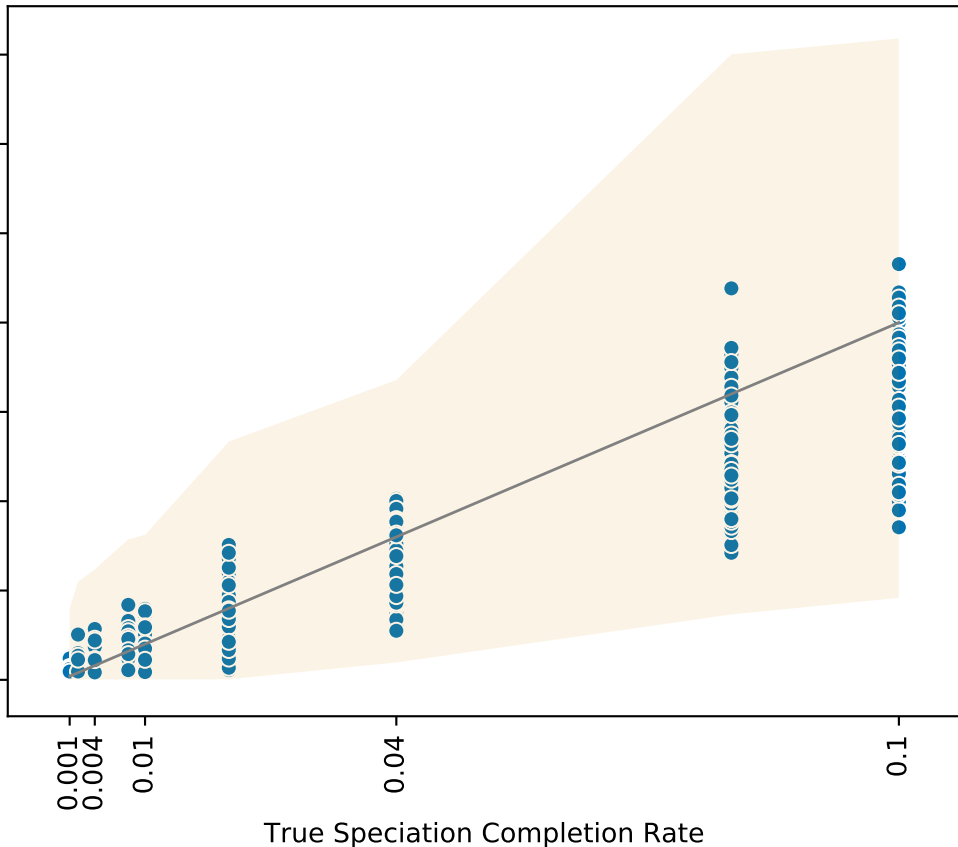

Supplement: S1 Data — This is an compressed archive that includes: scripts to simulate data, construct analysis pipelines, and cluster execution job files (delineate-performance-setup/bin); some notes on the parameter space we used (delineate-performance-setup/docs); scripts to collate, compile, and analyze results, as well as generate plots/figures from results data (delineate-performance-results/bin/); CSV/TSV files summarizing each replicate, including (true) parameters as well as inferred parameter values and probablities, as well as metadata such as analysis execution date/time, cluster location, etc. (delineate-performance-results/data/extracts); TSV files containing data simulation/generation logs, including random seeds etc. (delineate-performance-results/data/logs). Note that we omit the full (simulated) data sets due to size (> 5TB). However, these can be easily regenerated in identical detail using the same random seeds for the data generation (given in the “logs”) with the scripts found in the “setup” section above. Note also that the automatically generated logs provided above span a broad variety of studies and analyses, including not only the production runs reported here but also pilot runs, experimental studies, etc. Production run details relevant to this paper can be identified by correlating date/time/cluster with the information found in the “extracts” subdirectory above. (TBZ) [file pcbi.1008924.s002.tbz › delineate-performance-results/data/extracts/speciation-completion-rate/fig-speciation-completion-rate-results-single.pdf]

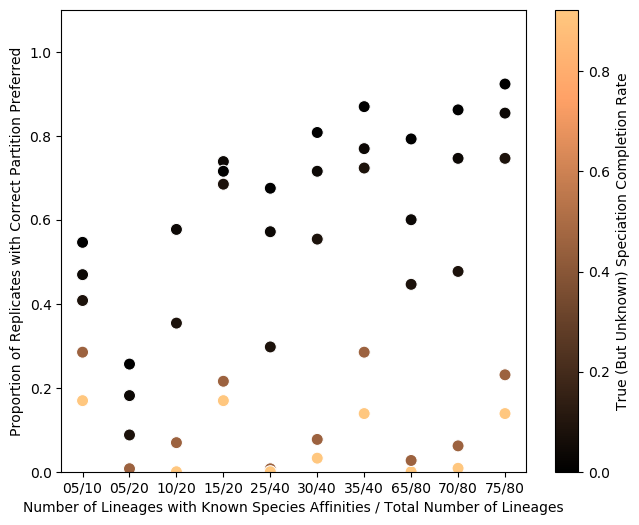

Supplement: S1 Data — This is an compressed archive that includes: scripts to simulate data, construct analysis pipelines, and cluster execution job files (delineate-performance-setup/bin); some notes on the parameter space we used (delineate-performance-setup/docs); scripts to collate, compile, and analyze results, as well as generate plots/figures from results data (delineate-performance-results/bin/); CSV/TSV files summarizing each replicate, including (true) parameters as well as inferred parameter values and probablities, as well as metadata such as analysis execution date/time, cluster location, etc. (delineate-performance-results/data/extracts); TSV files containing data simulation/generation logs, including random seeds etc. (delineate-performance-results/data/logs). Note that we omit the full (simulated) data sets due to size (> 5TB). However, these can be easily regenerated in identical detail using the same random seeds for the data generation (given in the “logs”) with the scripts found in the “setup” section above. Note also that the automatically generated logs provided above span a broad variety of studies and analyses, including not only the production runs reported here but also pilot runs, experimental studies, etc. Production run details relevant to this paper can be identified by correlating date/time/cluster with the information found in the “extracts” subdirectory above. (TBZ) [file pcbi.1008924.s002.tbz › delineate-performance-results/data/extracts/constrained-partition-estimation/fig-constrained-joint-partition-probabilities-scatter.png]

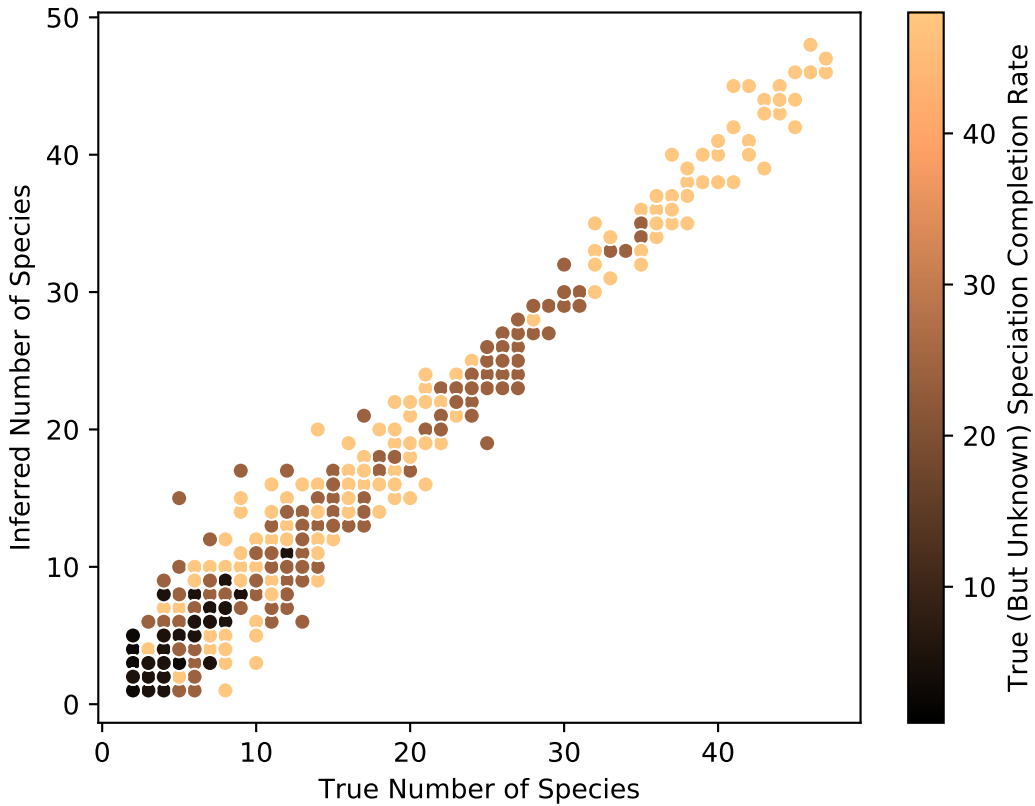

Supplement: S1 Data — This is an compressed archive that includes: scripts to simulate data, construct analysis pipelines, and cluster execution job files (delineate-performance-setup/bin); some notes on the parameter space we used (delineate-performance-setup/docs); scripts to collate, compile, and analyze results, as well as generate plots/figures from results data (delineate-performance-results/bin/); CSV/TSV files summarizing each replicate, including (true) parameters as well as inferred parameter values and probablities, as well as metadata such as analysis execution date/time, cluster location, etc. (delineate-performance-results/data/extracts); TSV files containing data simulation/generation logs, including random seeds etc. (delineate-performance-results/data/logs). Note that we omit the full (simulated) data sets due to size (> 5TB). However, these can be easily regenerated in identical detail using the same random seeds for the data generation (given in the “logs”) with the scripts found in the “setup” section above. Note also that the automatically generated logs provided above span a broad variety of studies and analyses, including not only the production runs reported here but also pilot runs, experimental studies, etc. Production run details relevant to this paper can be identified by correlating date/time/cluster with the information found in the “extracts” subdirectory above. (TBZ) [file pcbi.1008924.s002.tbz › delineate-performance-results/data/extracts/constrained-partition-estimation/data/pilot/fig-speciation-completion-rate-results-num-species-scatter.pdf]

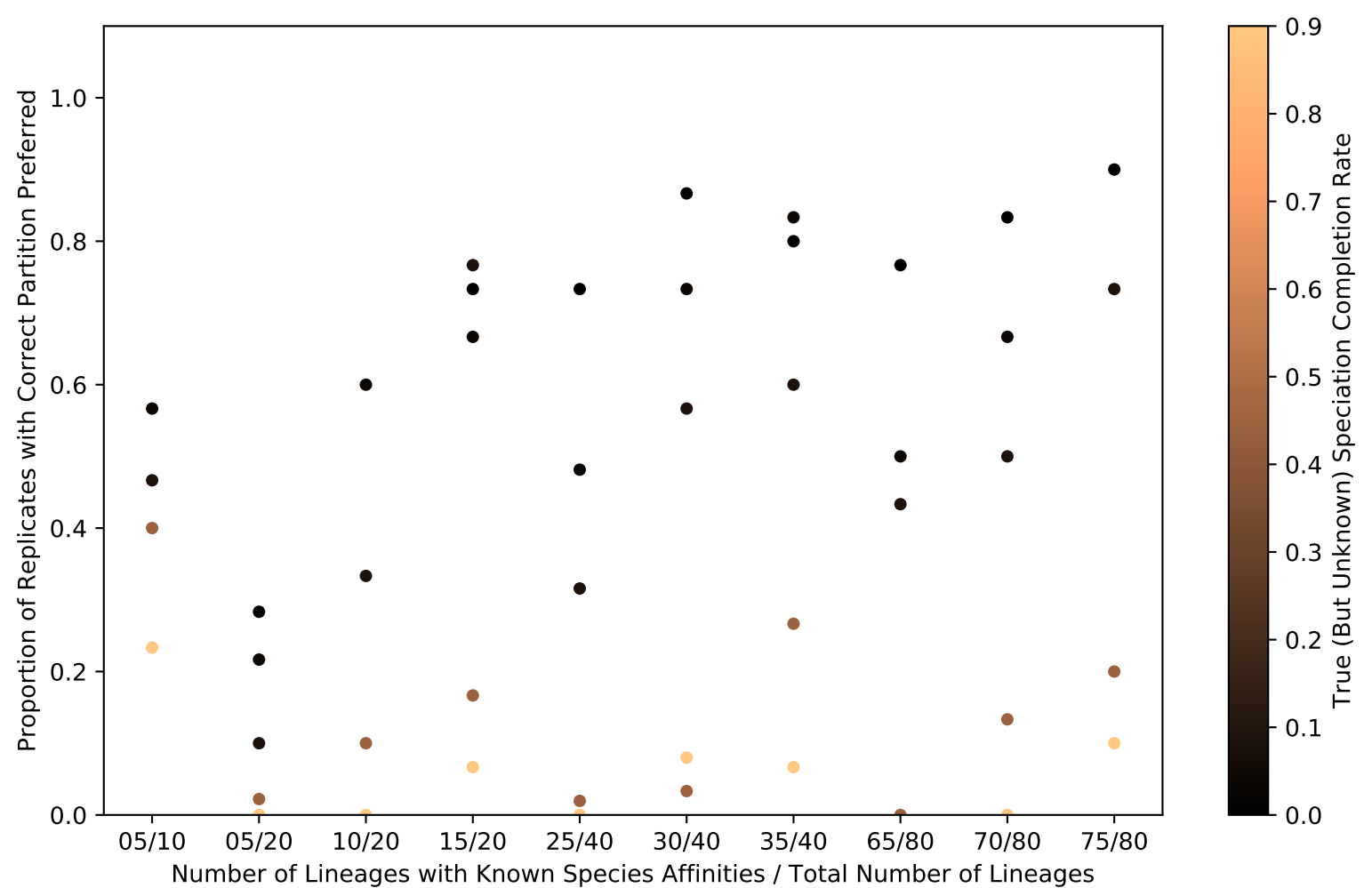

Supplement: S1 Data — This is an compressed archive that includes: scripts to simulate data, construct analysis pipelines, and cluster execution job files (delineate-performance-setup/bin); some notes on the parameter space we used (delineate-performance-setup/docs); scripts to collate, compile, and analyze results, as well as generate plots/figures from results data (delineate-performance-results/bin/); CSV/TSV files summarizing each replicate, including (true) parameters as well as inferred parameter values and probablities, as well as metadata such as analysis execution date/time, cluster location, etc. (delineate-performance-results/data/extracts); TSV files containing data simulation/generation logs, including random seeds etc. (delineate-performance-results/data/logs). Note that we omit the full (simulated) data sets due to size (> 5TB). However, these can be easily regenerated in identical detail using the same random seeds for the data generation (given in the “logs”) with the scripts found in the “setup” section above. Note also that the automatically generated logs provided above span a broad variety of studies and analyses, including not only the production runs reported here but also pilot runs, experimental studies, etc. Production run details relevant to this paper can be identified by correlating date/time/cluster with the information found in the “extracts” subdirectory above. (TBZ) [file pcbi.1008924.s002.tbz › delineate-performance-results/data/extracts/constrained-partition-estimation/data/pilot/fig-speciation-completion-rate-results-scatter.pdf]

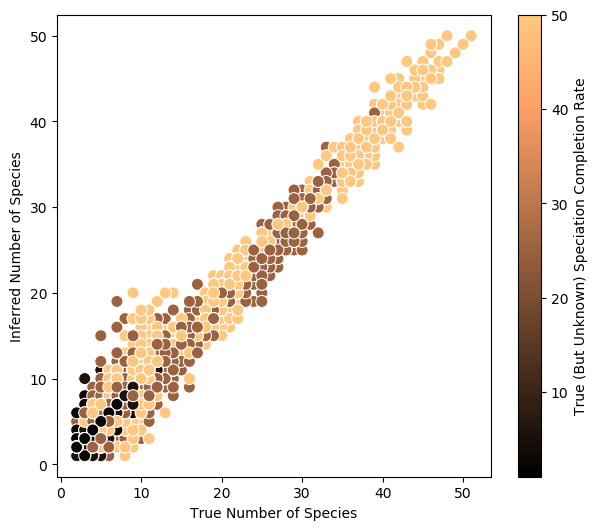

Supplement: S1 Data — This is an compressed archive that includes: scripts to simulate data, construct analysis pipelines, and cluster execution job files (delineate-performance-setup/bin); some notes on the parameter space we used (delineate-performance-setup/docs); scripts to collate, compile, and analyze results, as well as generate plots/figures from results data (delineate-performance-results/bin/); CSV/TSV files summarizing each replicate, including (true) parameters as well as inferred parameter values and probablities, as well as metadata such as analysis execution date/time, cluster location, etc. (delineate-performance-results/data/extracts); TSV files containing data simulation/generation logs, including random seeds etc. (delineate-performance-results/data/logs). Note that we omit the full (simulated) data sets due to size (> 5TB). However, these can be easily regenerated in identical detail using the same random seeds for the data generation (given in the “logs”) with the scripts found in the “setup” section above. Note also that the automatically generated logs provided above span a broad variety of studies and analyses, including not only the production runs reported here but also pilot runs, experimental studies, etc. Production run details relevant to this paper can be identified by correlating date/time/cluster with the information found in the “extracts” subdirectory above. (TBZ) [file pcbi.1008924.s002.tbz › delineate-performance-results/data/extracts/constrained-partition-estimation/fig-constrained-joint-partition-num-species-scatter.png]
