# Supplementary material for: Incorporating the speciation process into species delimitation
Source: PLoS Comput Biol. 2021 May 13;17(5):e1008924. doi: 10.1371/journal.pcbi.1008924 (PMC8118268; doi:10.1371/journal.pcbi.1008924)
Supplement: Attachment — Submitted filename: response-letter.pdf [file pcbi.1008924.s003.pdf]

Dear PLOS Biology,

We thank the editor and the anonymous reviewers for their time and effort in reviewing this manuscript, as well as for sharing their thoughtful comments, critiques, and suggestions for improvement. We have read through these latter very carefully and incorporated or otherwise responded to them thoroughly. In subsequent sections below, please find a detailed description of our responses to the remarks and suggestions of the editor and reviewers.

In addition, please note the following:

1. We have rerun all our simulations and analyses to take advantage of better logging facilities added to both our performance assessment scripts as well as the DELINEATE program itself. With these new logging facilities, we retain a extensive metadata in the results summaries, including random seeds, which allow for the *exact* regeneration of the datasets through the scripts we provide, *without* the need to download the original data (which exceed 5TB in size). As such, while, in addition to the performance assessment data generation and analyses scripts, we will provide spreadsheet summaries of our data with this submission (flatfile databases report parameters, inference results, and assessment of inference results, as well as the associated metadata such as dates, times, random seeds, execution hosts and directories, etc.), we are omitting inclusion of the full original data due to size considerations. We suggest that the data generation and analyses scripts in conjunction with the data summaries alone are sufficient to ensure full replicability of our studies. If the editors or the reviewers feel otherwise, we are happy to look into feasible ways that the original 5TB of data can be hosted and shared.

2. We are happy to report that our repeated analyses, which were carried out under different random seeds from the originals (in the first revision), produced results that are very similar and support the same conclusions. This speaks to us strongly of the robustness of our study results, as the reanalyzed data were very different in detail, being generated under different random seeds over the same points in parameter space. For the reference of the editor and reviewers, here we show comparisons of the results of the original vs. re-run analyses:

   - **Constrained Partition Estimation Analyses**

(Original) (Replicate)

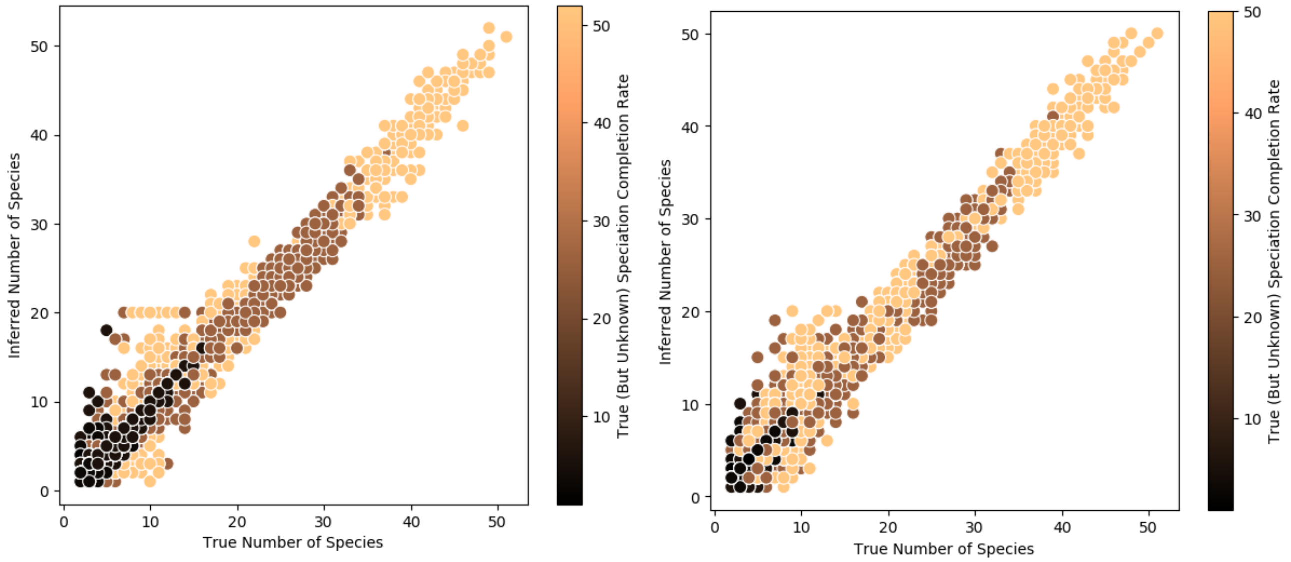

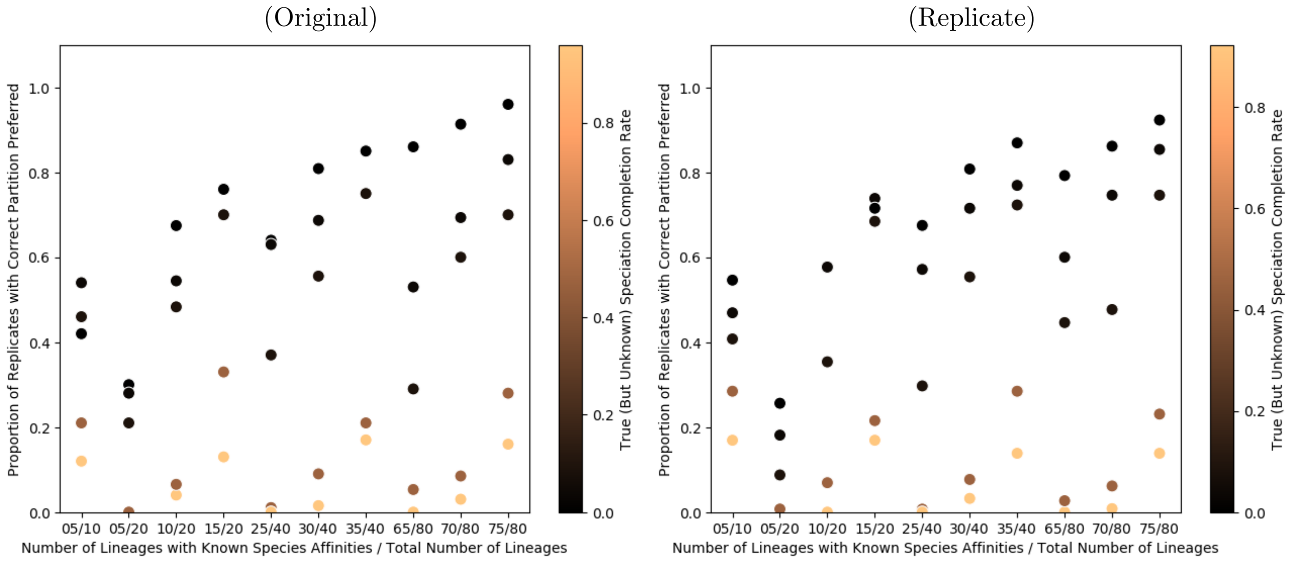

- **Speciation Completion Rate Performance Analyses**

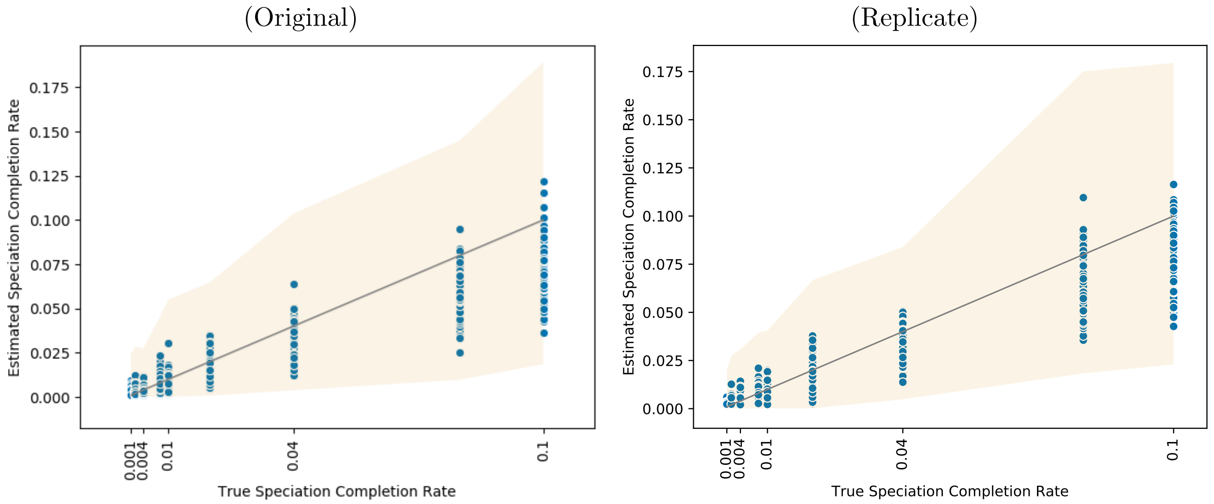

- **Unconstrained Partition Estimation Analyses**

**Statistic**	**Original Result**	**Replicate Result**
n	1000	1000
Mean number of true number species	4.94	5.092
Standard deviation of true number species	2.17	2.1217
Number of possible partitions per replicate	514, 229	514, 229
Proportion of replicates in which the true species partition was correctly inferred	0.008	0.012
Proportion of replicates in which the true species partition was in the 95% confidence interval	947 (94.7%)	925 (92.5%)

1 Responses to Editor Remarks

(1) The reviewers make an important point that it is not fully apparent to superficial readers of this manuscript that the model can only delimit species if some species are already defined. I think this should be clearly explained up front, returned to later, and apparent from the abstract.

We agree. We have added clarification of this in the abstract and introduction, and, of course, this is already discussed in detail later.

(2) Line 17-20. The fact that populations will be artificially promoted by MSC methods is independent of species concept, but what represents the correct level to define a species is very much dependent on species concept. A major absence in the introduction is defining what you mean by species – without that definition, it cannot be possible to objectively delimit them with a statistical analysis. I think you define them as "what systematists define them to be", i.e. that information external from the method is used to define enough species that you can estimate your parameters. I don't have a problem with that as a definition but it needs to be clearly stated. Without it, species have some mythical status in this approach and appear to be conjured from thin air, as nothing in the model actually specifies the correct level unless you know the parameter – which you can't do without knowing some species already! There is some useful discussion later on, but it needs to be much clearer upfront.

We added clarification that the species concept of the investigator is implictly captured by their specification of species identities of the subset of population lineages, rather than being provided or imposed by the model.

2 Responses to Reviewer #1 Remarks

I think incorporating the model of speciation process is a logical idea to correct the over-splits caused by the MSC-based delimitation. Also, this method can be useful not just for delimitation but for studies of speciation process itself. Nevertheless, I do not think that some of the authors claims in the manuscript is fully convincing and some corrections are required before publication. Especially, that the genomic sequences alone cannot correctly delimit species is not supported by their results. I think it is rather due to their specific model design.

The reviewer is correct that the inability to discriminate between population and species from genomic data alone is due to the model. However, this model here is the Multispecies Coalescent (MSC). What our model provides is essentially an extension to the MSC that, provided with additional (non-genomic) information in the form of systematic understanding of *some* species boundaries, the remaining species vs population boundary distinction can be inferred.

As already pointed out in the Leaché et al. 2018 paper, the protracted speciation (PS) model decouples speciation process and lineage splitting process. The PS model maps transition events on a population tree with a constant rate (sigma). This parameter is independent from the lineage branching process and is not directly affected by population processes like genetic differentiation or gene flow. Therefore, the inference of sigma is impossible with tree branch lengths alone. The species assignment of some tips is always required to infer the transition rate under this model setting. The manuscript should more clearly mention this property.

We have revised the text to note this.

In real situations, speciation process is very likely to be affected by multiple population level processes, and the transition rate likely correlated with these parameters (Such as Ne*m). Sigma probably could be estimated from them. Without testing the adequacy of the constant-independent-rate PS model, it is not safe to conclude that the delimitation is impossible from genomic data alone.

There are many possible ways that we might be able to estimate σ in the future from a variety of data, including

genomic. We look forward to a future in which these approaches are concretely formulated and developed. However, until such ways are actually developed and tested, we are unable to speak to them. Our statement regarding the limitations of genomic data to identifying species boundaries is with the specific context of our understanding of the problem today.

Line 51. The gdi thresholds in Jackson et al. and Leaché et al. are informed by biological observations. They set it to match with the observed species-population boundaries. This procedure is similar to estimating the transition rate from taxonomic knowledge used in this manuscript.

The gdi thresholds in Jackson et al. were indeed based on a small sample of particular empirical biological datasets. It is unclear how representative these are for the broader range of systems that investigators focus on, nor what the justification is (if any) in extending these threshold values to these systems, with very different biologies and demographic histories, from fungi to grasshoppers to shrews to elephants to dipterocarps. As such they remain heuristic rule-of-thumbs, with no clear way of assessing their fit or suitability for the specific data an investigator wishes to apply them to, nor any clear way of deriving appropriate thresholds when it is clear from the final results that they are *not* appropriate.

In contrast, our approach is more akin to, e.g., estimating the parameter of a model (e.g., the transition rates in a character substitution model), where a statistical estimate is made on data sampled from the specific system that is being studied, rather than leaving the user to make an arbitary choice from some general range.

Line 79. A brief model description is required here before reporting results.

Please note that we do actually provide a brief conceptual description of the model and how it works a few lines previosu to that referenced by the review, i.e., in lines 59 through 68. The full statisical description of the model is given in the "Materials & Methods" section. We followed the suggestions in the *PLOS Computational Biology* guidelines, in placing our "Materials & Methods" section after the "Discussion". If the reviewer recommends moving the "Materials & Methods" section to before the "Results" section, we are happy to do so if this is acceptable to the editor.

Line 92. "Figure 1" is missing in the text.

We have corrected this.

3 Responses to Reviewer #2 Remarks

(1) The entire model is not formally described anywhere in the manuscript, making it impossible for the reader to fully understand how the software works. The PBD model is described to some extent in Figure 1, but Figure 1 is not referenced anywhere in the manuscript. However, the parameter σ doesn't appear in the caption to Figure 1, although speciation completion is discussed there, and this parameter appears to be the key component of the model for this purpose, as it is discussed throughout the text. It seemed that perhaps the model would be carefully described in the section labeled "Statistical Model Description and Inference Algorithm", but it is not. It is stated that the data are sequences, and partitions of sequences into species are defined. But how, specifically, are the sequence data integrated with the PBD model? How is $Pr(\Lambda|\sigma, S)$ actually computed? It is imperative that the full model be carefully defined somewhere in the manuscript.

The model, as well as the inference algorithm, is described in the "Statistical Model Description and Inference Algorithm" section of the manuscript, under "Materials & Methods". The PBD model does not "see" the sequences, it "sees" the population tree which is the result of the MSC inference. The population tree is a result of the MSC inference that treats the sequence data. Similarly, $Pr(\Lambda|\sigma, S)$ describes the MSC model, and its computation is

carried out during the MSC population tree inference stage. Our original reference to this was not clear enough, limited to a citation of the MSC paper. We have now provided a more explicit explanation of this, including a new figure (Figure 4) that provides an overview of the entire workflow.

The authors also need to more carefully define the algorithm used in DELINEATE and/or specify what options were used in running the simulations. My guess is that all partitions are attempted, the likelihood is calculated on a fixed lineage tree for each partition, and the one with the highest probability is selected. If this is indeed correct, it should be stated.

This is correct, and this is we meant by reference to "maximum likelihood". The procedure is actually described in the "Constrained Species Delimitation" section: " ... and then calculate the probabilities of all possible partitions that include the species identities given the estimated speciation-completion rate. The different partitions can then be ranked according to their probabilities, with the partition of the highest probability constituting the maximum likelihood estimate of the delimited species boundaries. "

Also, how is a 95% confidence set obtained? Are partitions added in decreasing order of probability until 95% of the probability is accounted for? Or something else?

Exactly as stated by the reviewer – "partitions added in decreasing order of probability until 95% of the probability is accounted for". We have added this clarification to the text.

(2) Is the species completion rate underestimated in these types of models because some species die out? It might be good to comment on this.

This is probably rather due to saturation – multiple speciation completion events on a single branch result in the same number of descendent species in the data. We have added clarification regarding this.

(3) For the unconstrained simulations, is there any pattern in the kinds of partitions that had the highest probability? Does the method tend to underestimate or overestimate the number of species? Are certain scenarios less likely to be detected than others?

This study design randomized over broad patterns of partitions to maximize coverage parameter space while integrating out uncertainty or artifacts of partition configuration. It would certainly be interesting to explore specific biases or trends based on, e.g., topologically-constrained partitions (i.e., where an entire a subtree is unknown), but that would require focused studies due to the computational demand. As such, these are excellent directions for future studies.

(4) The first sentence of the Discussion doesn't seem to be to be supported directly by any of the analyses. Is there evidence presented that this method prevents oversplitting, or is this comment just based on the fact that the model is expected to do that?

Please reference Figure 2(a) which shows the numbers of species inferred under this model broadly tracks the true number of species. Compare this performance to that of the classical Multispecies Coalescent as seen in Figure 2(a) of Sukumaran and Knowles, 2017, which show the oversplitting, as inferred number of species are greatly inflated with respect to number of true species due to conflation of within-species lineages boundaries with species boundaries.

Similarly, while I don't necessarily disagree with the claim (lines 152-153) that ".. inferences that rely on genetic data alone, without reference to any other information for delimiting species, are not reliable", I don't see that this claim is justified by the work presented here. Just because this model doesn't allow for accurate delimitation doesn't mean that there is no model for genetic data that can't produce an accurate delimitation.

We have revised the statement to the more precise "*Multispecies Coalescent species delimitation* inferences that rely on genetic data alone ...".

- line 27, "for their particular" — complete the phrase

Fixed.

- lines 35-39, problem with the latex citations

Fixed.

- line 44, maybe remove "the" before "MSC-based"

Fixed.

- line 58, remove the comma after "divergence"

Fixed.

- line 141, "under at reasonable" — re-word

Fixed.

- line 157, combine the first two paragraphs of this section

Done.

- line 214, missing punctuation

Fixed.

- line 255, "any" is repeated

Fixed.

- line 264, "as it is necessary assumption" — re-word

Fixed.

- first paragraph of Materials & Methods reads awkwardly — might be better to say something like "DELINEATE" has 3 modes of inference: . . . "

Fixed.

- Section on "Constrained Species Delimitation", this is very difficult to read since the model has not yet been introduced. What does the parameter σ mean?

We have added an explanation of this parameter. Alternatively, we could begin the Materials & Methods section with an model description.

- line 394, extra semi-colon

Fixed.

- paragraph starting on line 383, I found this redundant/unnecessary

We think the paragraph serves to usefully communicate a conceptual understanding of the role of the systematic information an investigator provides. By establishing the analogy with a Bayesian prior, which many investigators are familiar with in practice even if not the deep theoretical sense, it provides guidance on how to think about this systematic information, both in the way that it could be provided as well as in the way it could influence results.

-line 413, refers to the PBD model, not yet introduced

The PBD model is introduced earlier in the paper and in Figure 1. Note that this work does not itself present or describe the PBD model, but incorporates it into the speciation delimitation model. The PBD model has been extensively discussed and developed in a line of other work by other authors, and it is out of the scope of this paper to describe it beyond the brief description that we provide (we provide both a intuitive understanding of the model as well as a summary of its expression). We provide citations to the PBD model in the paper, and in this particular line, provide a citation to the statement we make regarding the lack of analytical solution for the speciation-completion rate.

Again, however, we could move the statistical model description to the beginning of the Materials & Methods section to address this issue.

- line 444, "speciation" is misspelled

Fixed.

- line 451, "the" is repeated

Fixed.

- line 505, "partition" -¿ "partitions"

Fixed.

- Caption to Fig. 3, "minimum" is misspelled

Fixed.

- When referencing the journal Systematic Biology, capitalize both words.

Fixed.

Have all data underlying the figures and results presented in the manuscript been provided? Large-scale datasets

should be made available via a public repository as described in the PLOS Computational Biology data availability policy, and numerical data that underlies graphs or summary statistics should be provided in spreadsheet form as supporting information.

The entire raw data exceeds 5 TB in size. This is not feasible to host in any public repository. However, our study design and implementation means that we do not need to actually refer to the raw data for validation and replicability. All data is generated by scripts (which we do provide) which take random seed values. These random seed values ensure full re-generation of the raw data we use.

While re-generation of the specific random data that we generated might be useful for validation of our *reporting*, validation of our *results* (and thus the conclusions that rest on these) is better done by repeating the analyses using different random seed. In fact, we did exactly the latter, taking advantage of better logging facilities in both our study script as well as the DELINEATE program, and found almost identical results. The summaries of these results (CSV files, with each row reporting the parameters, inference results, and inference assessments for a single data point) will be included with this submission as well as made publically available.
