# Supplementary material for: Incorporating the speciation process into species delimitation
Source: PLoS Comput Biol. 2021 May 13;17(5):e1008924. doi: 10.1371/journal.pcbi.1008924 (PMC8118268; doi:10.1371/journal.pcbi.1008924)
Supplement: Attachment — Submitted filename: response-letter2.pdf [file pcbi.1008924.s004.pdf]

Dear PLOS Biology,

We thank the editor and the anonymous reviewers for their time and effort in reviewing our revision. Below, please find the reviewer comments and our responses:

1. Also, I would recommend moving the Materials&Methods before the Results. In the current version of manuscript, it is very hard to understand the Results section because no information about the modes of software and simulation setups is provided beforehand. (I asked them to add some sentences at the beginning of the Results, but they did not.) I think moving the M&M to the front is easier and PLOS computation biology's style looks flexible according to their guideline.

   We apologize for failure to add clarifying sentences to the beginning of the results. We agree entirely that moving the "Materials and Methods" is, however, the ideal solution, and this has been done in this revision.

2. Providing an equation of $Pr(Lambda|sigma, S)$ will help readers to understand this method. If I understand it correctly, its actual form is $Pr(No.transitionevents > 0|sigma, branchlength)$ or $Pr(No.transitionevents == 0|sigma, branchlength)$ assuming Poisson-distributed events and branch lengths are determined by S and Lambda. The calculation of an overall likelihood and dynamic programming can be clearly understood with an explicit equation too.

   We have added the requested equations in the main text, as well as provided a full description of the general dynamic programming algorithim in the Supplemental Information.

3. It is a bit confusing to use "likelihood" and "probability" interchangeably. I think that $Pr(Lambda|sigma, S)$ is a likelihood function of partition, Lambda, and the algorithm calculate a likelihood of delimitation/partition.

   We have rephrased the text to reflect this.
